# Bee Attack or Heart Attack: Kounis Syndrome

**DOI:** 10.7759/cureus.14740

**Published:** 2021-04-28

**Authors:** Kashmala Khan, Gabor Szalai, Humayun Anjum, Francis Dimtri, Deanna Yamamura, Salim Surani

**Affiliations:** 1 Internal Medicine, Corpus Christi Medical Center, Corpus Christi, USA; 2 Interventional Cardiology, Corpus Christi Medical Center, Corpus Christi, USA; 3 Pulmonary/Critical Care Medicine, Corpus Christi Medical Center, Corpus Christi, USA; 4 Cardiology, Corpus Christi Medical Center, Corpus Christi, USA; 5 Internal Medicine, University of North Texas, Dallas, USA

**Keywords:** kounis syndrome, anaphylactic reaction, allergic acute coronary syndrome, plaque rupture, allergic angina, stent thrombosis, coronary artery vasospasm

## Abstract

Kounis syndrome (KS) is defined as an allergic or hypersensitivity reaction leading to coronary vasospasm and acute coronary syndrome. The inflammatory mediators released during the body’s reaction to an allergen causes vasoconstriction, plaque rupture, platelet aggregation, and even thrombosis of an existing coronary stent. Over the years, many allergens including drugs, environmental exposures, and animal and insect bites have been implicated in KS. Patients may present with elevated cardiac enzymes and electrocardiographic changes. We describe a case of a patient with no prior cardiac history who presented to the emergency department seeking treatment after multiple bee stings. The patient had non-specific electrocardiogram (ECG) changes and elevated cardiac enzymes consistent with a non-ST-elevation myocardial infarction. The patient underwent a pharmacologic stress test and myocardial perfusion imaging, which showed a perfusion defect consistent with ischemia. Selective right and left coronary angiography revealed a critical lesion at the proximal left circumflex artery. This was managed with percutaneous coronary intervention utilizing a bare-metal stent.

## Introduction

Kounis syndrome (KS) describes the association of acute coronary syndrome (ACS) with concurrent anaphylactic or hypersensitivity disorder. The syndrome described first by Kounis in 1991, as “allergic angina” refers to a hypersensitivity or anaphylactoid reaction to an allergen that can lead to cardiovascular signs and symptoms [1]. There is inadequate data regarding the exact incidence and prevalence of KS. According to one cohort study, the incidence of KS in hospitalized patients with hypersensitivity or anaphylactic reactions was 1.1% [2]. After exposure to an allergen, there is activation of mast cells with concurrent interaction of T-lymphocytes and macrophages. The underlying mechanism of KS involves release of inflammatory cytokines that can exert their effect on multiple organ systems. Some of the mediators released during the degranulation of mast cells include histamine and arachidonic acid products such as leukotrienes, thromboxane, prostaglandins, tryptase, and chymase [3]. The release of these inflammatory mediators can lead to vasoconstriction of the coronary arteries, plaque eruption, and platelet aggregation. Various drugs and environmental exposures have been implicated to cause KS. There are three variants of KS. Type 1 KS is seen in patients with coronary artery vasospasm caused by the underlying anaphylactic reaction but no prior history of atherosclerotic coronary artery disease (CAD). Patients with type 2 KS have pre-existing quiescent atheromatous plaques that may rupture with the release of inflammatory mediators leading to an acute myocardial infarction (MI). Type 3 KS refers to the sub-type with stent thrombosis. Patients can be seen with electrocardiogram (ECG) changes and elevated cardiac enzymes. Reported ECG changes include ST elevations or depressions, heart block, and various cardiac arrhythmias [4]. According to one study, KS has been seen in 1.1% of anaphylactic or hypersensitivity reactions among hospitalized patients [5]. Non-ST-elevation MI was most prevalent and seen in 0.7% patients followed by 0.2% with unstable angina and 0.2 % with ST-elevation MI. In addition, KS was associated with a high inpatient mortality rate of 7% [5]. Here we present an interesting case of a patient diagnosed with and treated for KS secondary to bee stings.

## Case presentation

A 69-year-old male with a past medical history significant for diabetes mellitus, essential hypertension, hyperlipidemia, chronic kidney disease stage 3, and benign prostatic hypertrophy presented to the emergency department (ED) via ambulance after a bystander noticed him to have suffered multiple bee stings. The patient was performing yardwork, mowing a lawn with a tractor, and bumped into an abandoned house when a swarm of bees attacked him. He suffered multiple bee stings and managed to escape in his tractor. Upon initial evaluation in the ED, he complained of feeling unwell, with sensation of his throat closing, nausea, and a rash from the bee stings. During this time, the patient denied chest pain, discomfort, dyspnea on exertion, exertional intolerance, or any previous cardiac history. Physical examination was significant for raised erythematous lesions located at the scalp, face, neck, and hands. There was no evidence of respiratory compromise. His home medications included metformin, glipizide, lisinopril, and tamsulosin.

**Figure 1:**
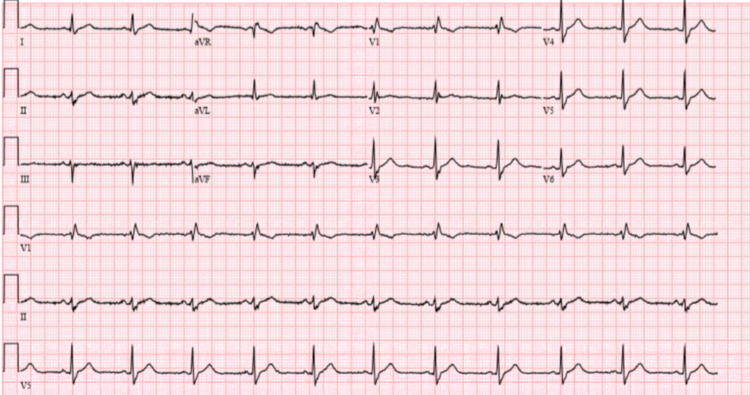
Initial ECG in the emergency room with non-specific ST changes.

Vital signs showed an initial blood pressure of 134/70 mmHg, heart rate of 98 beats per minute, and oxygen saturation of 92% on room air. Pertinent labs included complete blood count, with a white cell count of 17,260/uL, hemoglobin of 18.3 g/dL, and platelet count of 319,000/uL. His initial basic metabolic panel showed acute kidney injury with creatinine of 2.78 mg/dL and GFR (glomerular filtration rate) of 23. The ECG revealed a normal sinus rhythm at a rate of 70 bpm, left axis deviation, right bundle branch block, and left anterior fascicular block with a QRS duration of 130 ms; no significant ST-T wave segment changes were identified (Figure 1).

**Figure 2:**
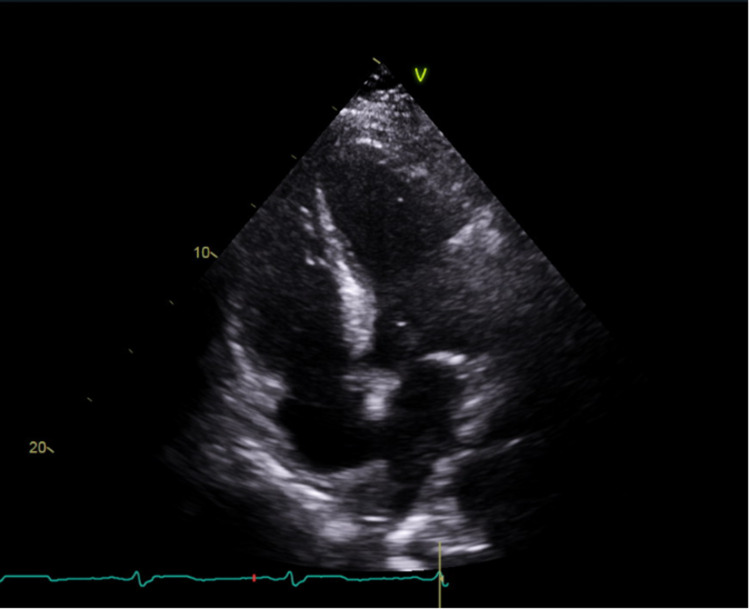
Transthoracic echocardiogram showing a four-chamber view, with no wall motion abnormalities seen.

He received intravenous (IV) fluids, IV methylprednisolone, and IV diphenhydramine in the ED. His initial troponin-I was negative at <0.04, repeat testing showed that it had increased to 1.05 ng/mL and 1.33 ng/mL and then trended down to 0.65 ng/mL and 0.35 ng/mL. He was also assessed by the critical care team. He was transferred to the ICU for further management. Cardiology was consulted for possible ACS. He was given a loading dose of aspirin, clopidogrel, high-intensity statin, and full-dose low-molecular-weight heparin. His symptoms of nausea and throat tightness were thought to be angina equivalent symptoms. An echocardiogram was performed, which showed no wall motion abnormalities, hyperdynamic left ventricular systolic function with an estimated ejection fraction (EF) of > 70%, and no evidence of diastolic dysfunction (Figure 2).

**Figure 3:**
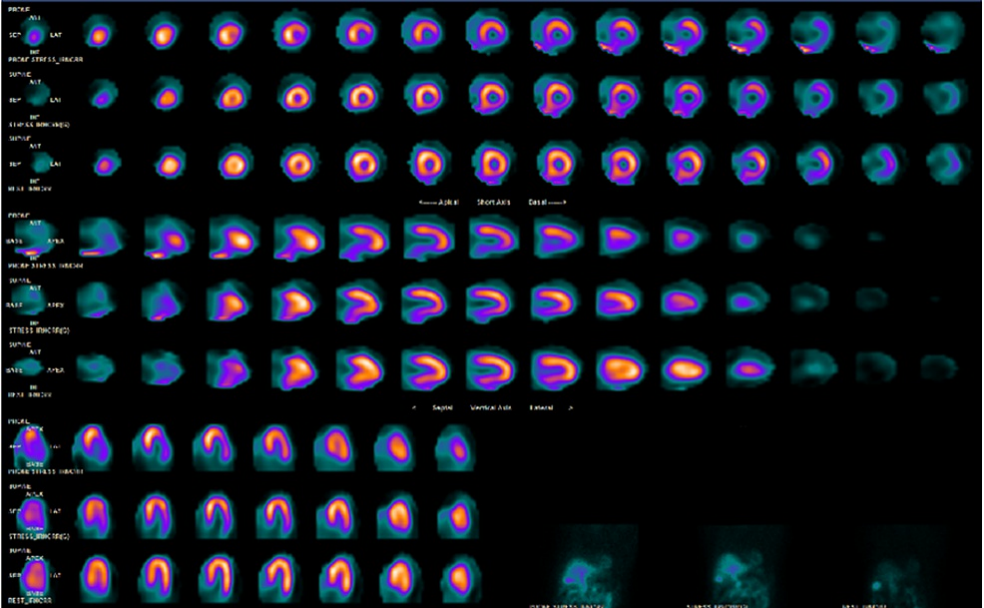
Myocardial perfusion imaging with the defect seen both in prone and supine

On day 2 of hospitalization, he underwent a pharmacologic stress test with myocardial perfusion imaging. His vital signs remained stable during the pharmacological stress test and recovery. ECG showed at baseline normal sinus rhythm with a right bundle branch block. During the pharmacological stress test, sinus tachycardia with nonspecific ST-T wave changes was present. There was no pathological extra-cardiac uptake and no significant patient motion artifact. There was a moderately sized defect with moderate-to-severe reversibility in the basal to mid-inferior and infero-lateral regions suggestive of ischemia. This defect was not corrected with prone images (Figure 3).

**Figure 4:**
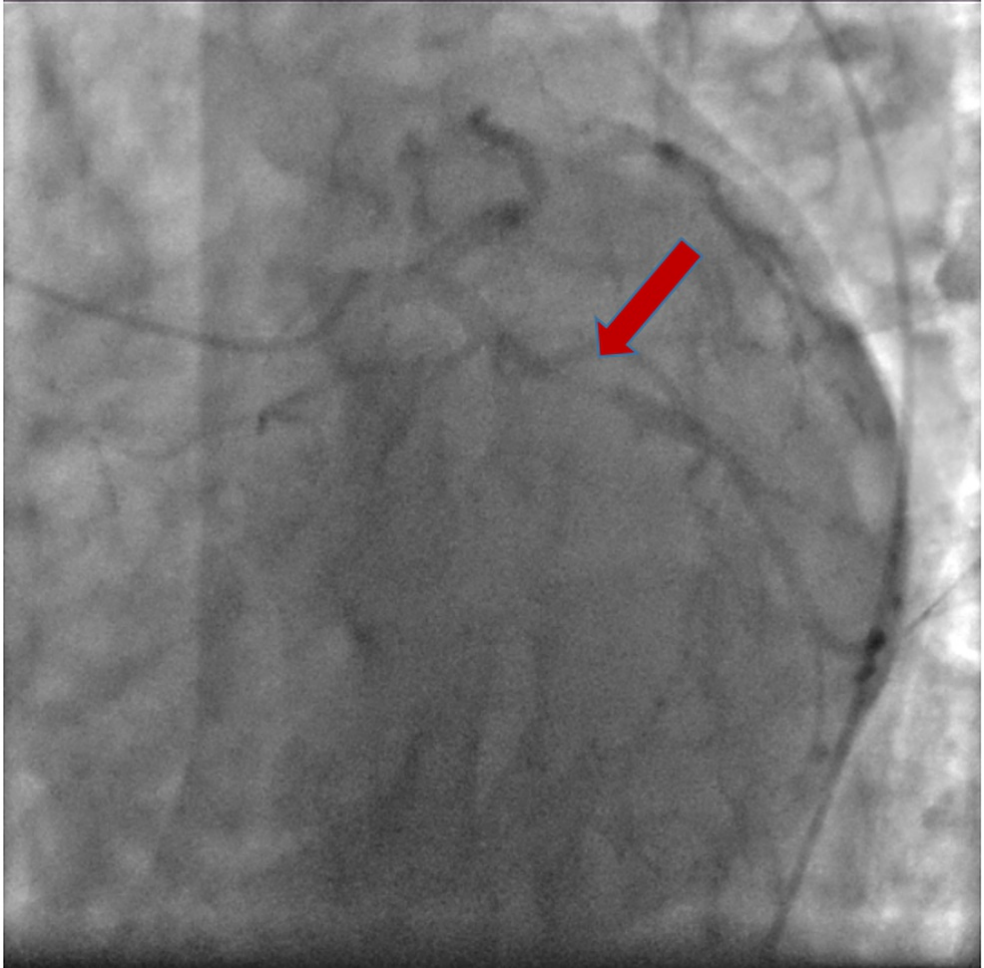
Image showing proximal left circumflex artery with 95% stenosis (red arrow)

**Figure 5:**
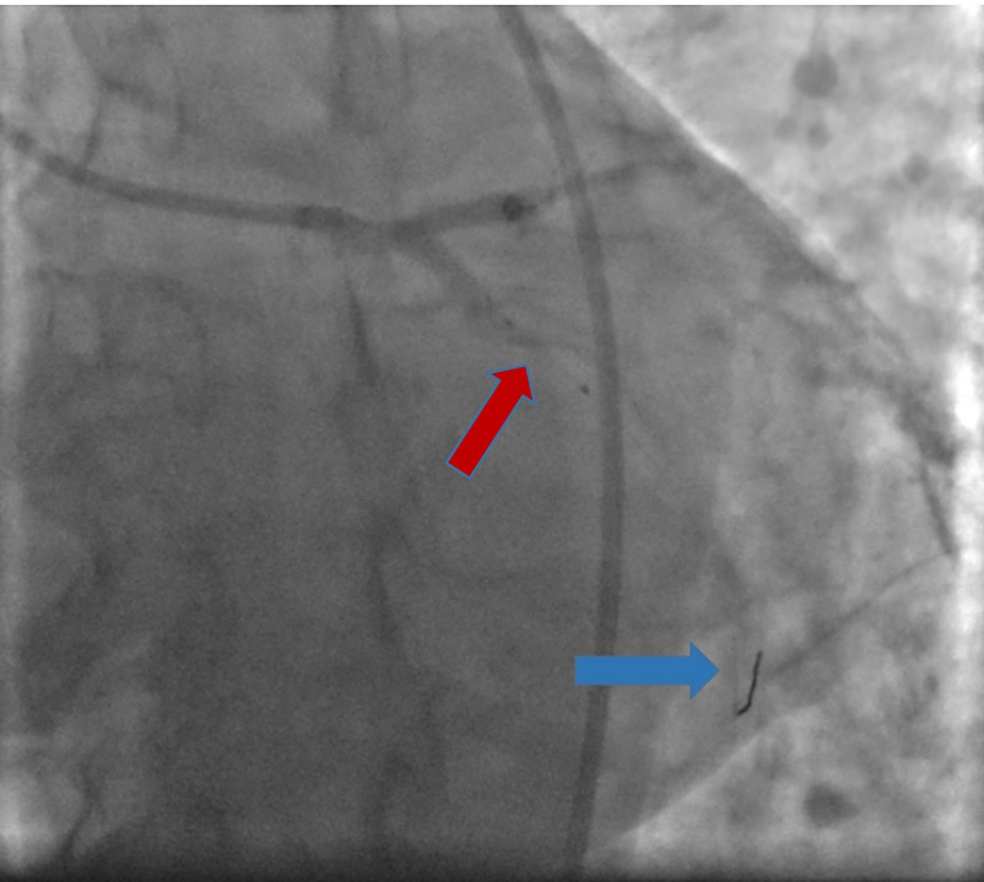
Image showing stent in the left circumflex artery (red arrow) and the tip of the wire (blue arrow)

**Figure 6:**
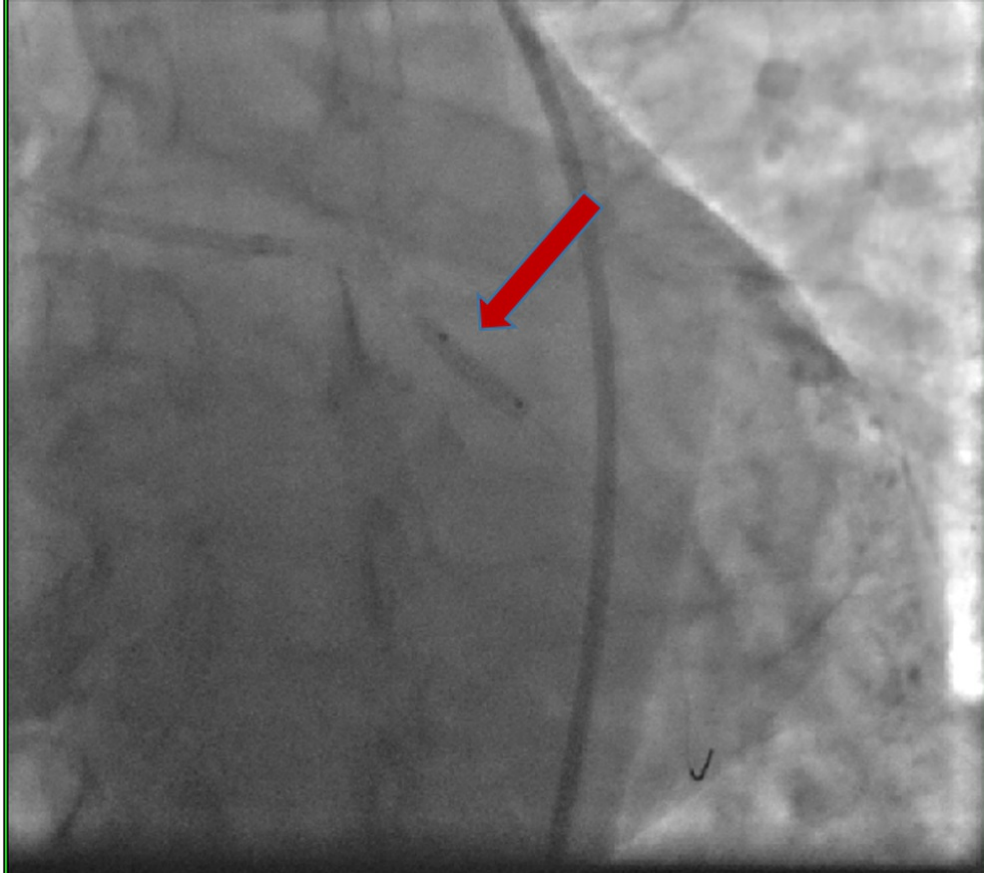
Image showing the deployed stent (red arrow)

**Figure 7:**
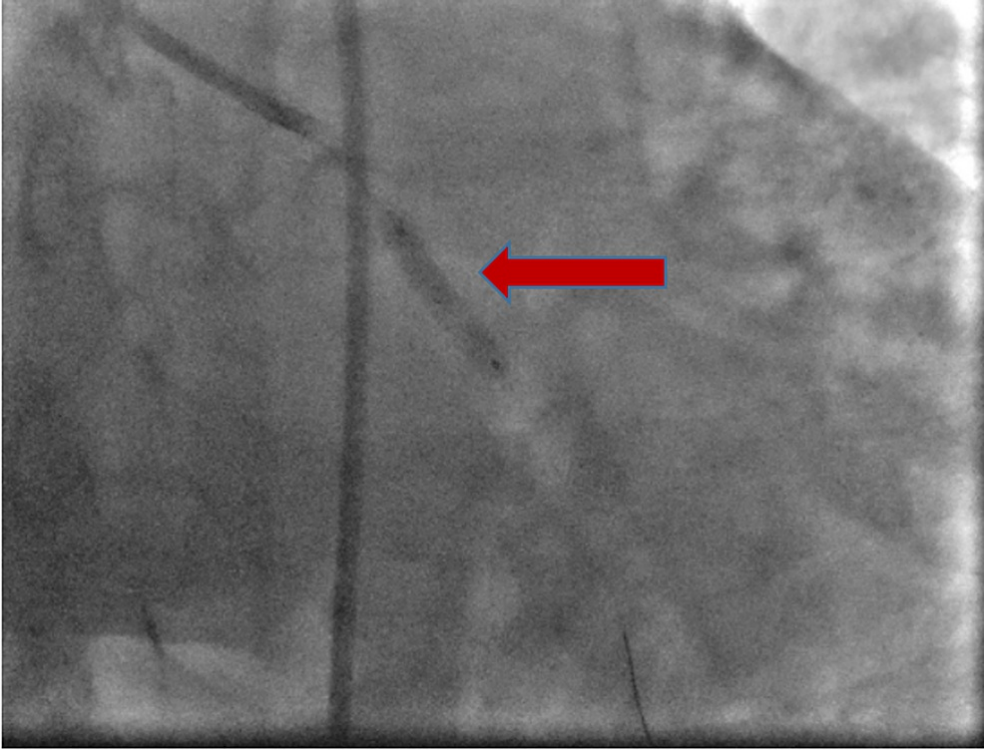
Image showing coronary angioplasty after the dilatation of the balloon (red arrow)

**Figure 8:**
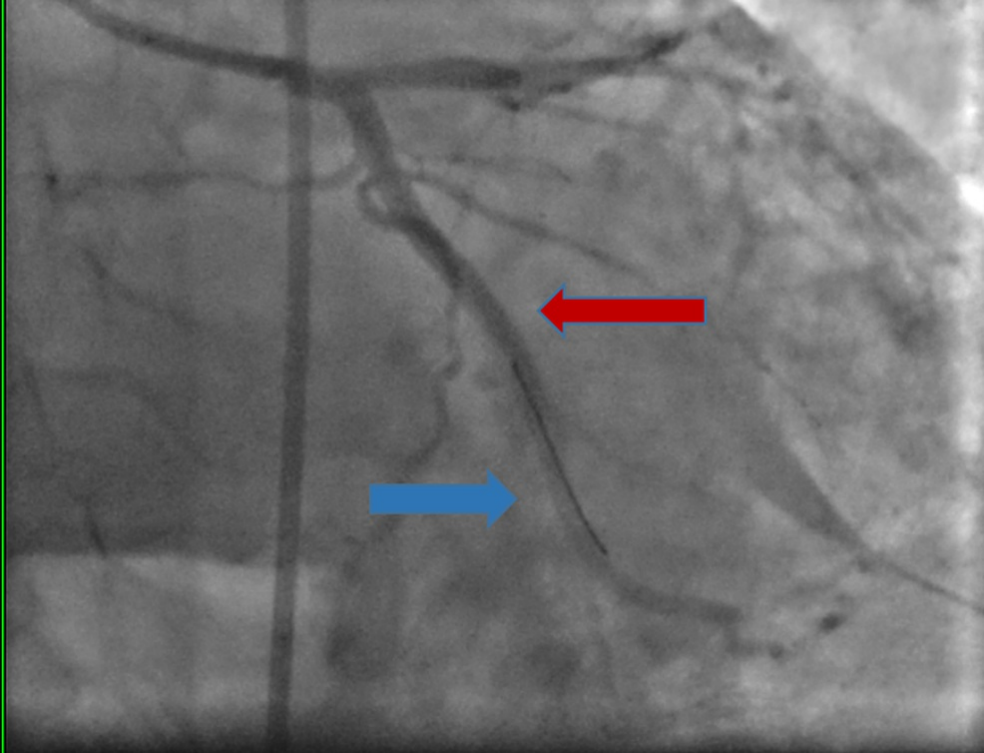
Angiogram showing stent in the left circumflex artery (red arrow) and the tip of the wire in place (blue arrow)

The patient’s creatinine remained elevated, and the nephrology service was consulted for the management of acute kidney injury. After improvement of his renal function with IV hydration, selective right and left angiography was performed revealing 95% stenosis of the proximal left circumflex and 20% stenotic plaque in the left main and right coronary arteries. The patient subsequently underwent angioplasty and stenting with a bare-metal 3.0 x 15-mm stent (Figures 4-8).

The patient tolerated the procedure well and resumed aspirin and clopidogrel therapy. Outpatient follow-up with cardiology was arranged.

## Discussion

KS, once a rare entity, is now increasingly recognized by clinicians as a potential cause for ACS [2]. Although KS has been encountered worldwide, it has been more frequently seen and reported in countries such as Greece, Italy, Spain, and Turkey. This could partly be due to specific environmental factors such as climate, frequency of animal bites, and possibly increased awareness of physicians [3]. KS is the association between an allergic reaction that can lead to vasospastic angina and possible MI secondary to mast cell activation and release of various inflammatory mediators. Although KS has been most notably linked to coronary vasospasm, there is increasing evidence that the effects extend to other organ systems too. The vasospasm can be seen in the arterial supply to the gastrointestinal tract causing mesenteric ischemia or to the cerebral circulation leading to ischemia or infarction. Common offending agents include medications such as non-steroidal anti-inflammatory drugs (NSAIDs), antibiotics, animal or insect bites, bee stings, contrast exposure, and reaction to seafood or shellfish [1].

Managing KS requires a multidisciplinary approach. The first step is to remove the offending agent to prevent further formation and propagation of the inflammatory mediators. The patient’s cardiac and allergic symptoms need to be treated simultaneously. To suppress the allergic reaction, IV fluids, steroids, and anti-histamines can be given. Nitrates and calcium channel blockers can be given for coronary vasospasm. Morphine, which is commonly given for chest pain during an ACS, has the ability to potentially increase the release of histamine and worsen the underlying vasospasm and should be avoided in patients with KS. Other medications to avoid include beta-blockers and epinephrine. Beta-blockers, while used in ACS, may lead to unopposed alpha-adrenergic activity and worsening vasospasm. Epinephrine, on the other hand, is widely used for anaphylactic reactions but must be cautiously used in KS as it may paradoxically worsen vasospasm [2].

The three aforementioned variants of KS are vasospasm in the absence of established CAD, vasospasm in the presence of atheromatous disease (likely due to plaque rupture), and stent thrombosis. Our patient likely can be classified as having the type 2 variant of KS. He had risk factors for CAD and evidence of mild atherosclerotic disease seen on heart catheterization. It is important to recognize patients with risk factors for cardiovascular disease even if they do not have established CAD. Similarly, ACS and KS should be considered as a possibility in patients presenting with a hypersensitivity reaction. The underlying pathophysiology of KS is vasospasm, and many patients have signs of vasospasm such as angina or angina equivalent symptoms [3].

## Conclusions

KS occurs more frequently than reported and should be considered in patients presenting with ACS and type 1 hypersensitivity reactions. Increased awareness regarding this clinical entity may give rise to prompt diagnosis and treatment. Patients with KS may not have cardiac symptoms despite significant vasospasm, but they should be investigated thoroughly.
